# Lung function decline preceding chronic respiratory failure in spinal muscular atrophy: a national prospective cohort study

**DOI:** 10.1186/s13023-023-02634-4

**Published:** 2023-02-23

**Authors:** Esther S. Veldhoen, Camiel A. Wijngaarde, Ruben P. A. van Eijk, Fay-Lynn Asselman, Negina Seddiqi, Louise A. M. Otto, Marloes Stam, Inge Cuppen, Renske I. Wadman, Roelie M. Wösten van Asperen, Erik H. J. Hulzebos, Laura P. Verweij van den Oudenrijn, Bart Bartels, Jasmijn Boezer, M. Gaytant, Cornelis K. van der Ent, W. Ludo van der Pol

**Affiliations:** 1 Department of Pediatric Intensive Care, Wilhelmina Children’s Hospital, University Medical Center Utrecht, Utrecht University, PO Box 85090, 3508 AB Utrecht, The Netherlands; 2 Department of Neurology, UMC Utrecht Brain Center, University Medical Center Utrecht, Utrecht University, Utrecht, The Netherlands; 3 Biostatistics and Research Support, Julius Center for Health Sciences and Primary Care, University Medical Center Utrecht, Utrecht University, Utrecht, The Netherlands; 4 Child Development and Exercise Center, Wilhelmina Children’s Hospital, University Medical Center Utrecht, Utrecht University, Utrecht, The Netherlands; 5 Center of Home Mechanical Ventilation, Department of Pulmonology, University Medical Center Utrecht, Utrecht University, Utrecht, The Netherlands; 6 Department of Pediatric Pulmonology, Wilhelmina Children’s Hospital, University Medical Center Utrecht, Utrecht University, Utrecht, The Netherlands

**Keywords:** Lung function, Natural history, Neuromuscular, Respiratory failure, Spinal muscular atrophy

## Abstract

**Background:**

Progressive lung function decline, resulting in respiratory failure, is an important complication of spinal muscular atrophy (SMA). The ability to predict the need for mechanical ventilation is important. We assessed longitudinal patterns of lung function prior to chronic respiratory failure in a national cohort of treatment-naïve children and adults with SMA, hypothesizing an accelerated decline prior to chronic respiratory failure.

**Methods:**

We included treatment-naïve SMA patients participating in a prospective national cohort study if they required mechanical ventilation because of chronic respiratory failure and if lung function test results were available from the years prior to initiation of ventilation. We analyzed Forced Vital Capacity (FVC), Forced Expiratory Volume in 1 s (FEV_1_), Peak Expiratory Flow (PEF) and Maximum Expiratory Pressure (PE_max_). We studied the longitudinal course using linear mixed-effects models. We compared patients who electively started mechanical ventilation compared to patients who could not be weaned after acute respiratory failure.

**Results:**

We analyzed 385 lung function tests from 38 patients with SMA types 1c–3a. At initiation of ventilation median age was 18.8 years (IQR: 13.2–30.1) and median standardized FVC, FEV_1_ and PEF were 28.8% (95% CI: 23.5; 34.2), 28.8% (95% CI: 24.0; 33.7) and 30.0% (95% CI: 23.4; 36.7), with an average annual decline of 1.75% (95% CI: 0.86; 2.66), 1.72% (95% CI: 1.04; 2.40) and 1.65% (95% CI: 0.71; 2.59), respectively. Our data did not support the hypothesis of an accelerated decline prior to initiation of mechanical ventilation. Median PE_max_ was 35.3 cmH_2_O (95% CI: 29.4; 41.2) at initiation of mechanical ventilation and relatively stable in the years preceding ventilation. Median FVC, FEV_1,_ PEF and PE_max_ were lower in patients who electively started mechanical ventilation (*p* < 0.001).

**Conclusions:**

Patterns of lung function decline cannot predict impending respiratory failure: SMA is characterized by a gradual decline of lung function. We found no evidence for an accelerated deterioration. In addition, PE_max_ remains low and stable in the years preceding initiation of ventilation. Patients who electively started mechanical ventilation had more restrictive lung function at initiation of ventilation, compared to patients who could not be weaned after surgery or a respiratory tract infection.

## Background

Hereditary proximal spinal muscular atrophy (SMA) is primarily characterized by progressive weakness of axial, proximal limb and respiratory muscles. Respiratory complications are among the most prevalent and can be life threatening [1, 2]. Natural history studies have shown that lung function and respiratory muscle strength deteriorate in treatment-naïve patients with early onset SMA, i.e. types 1, 2 and 3a [3–7]. It may remain relatively stable in late onset SMA types 3b and 4 [7]. The majority of treatment-naïve patients with SMA types 1 and 2 eventually need mechanical ventilation [1, 3, 8]. Ventilation risk is associated with SMA type and therefore with achieved motor milestones [8]. There is consensus that patients with SMA types 2 and 3 with symptomatic nocturnal hypoventilation or daytime hypercarbia should start home mechanical ventilation to correct hypoventilation and associated symptoms [1, 9].

The current standards of care propagate early assessments of lung function and use of supportive respiratory care in patients with SMA, specifically in types 1-3a. This includes mechanical ventilation, physiotherapy, air stacking, or mechanical insufflation-exsufflation techniques [1]. Although the natural history of lung function has been studied in the past years, its decline in the years prior to initiation of mechanical ventilation in patients with SMA has not been studied in detail. Such data would help to steer clinical decision making with regards to respiratory care, facilitate timing of counseling for impending chronic respiratory failure and may also be helpful for the evaluation of effects of newly introduced SMA therapies [10].

Therefore, we longitudinally assessed how lung function and expiratory muscle strength change in the years preceding chronic respiratory failure with initiation of (nocturnal) mechanical ventilation in treatment-naïve patients with SMA. We hypothesized that lung function would decline more steeply prior to initiation of mechanical ventilation, in accordance with the increases of carbon dioxide levels we described previously [11].

## Materials and methods

Patients enrolled in this study participate in an ongoing national prospective cohort study on SMA [6–8]. Patients with genetically confirmed SMA were included if they required (nocturnal) mechanical ventilation because of chronic respiratory failure and if lung function test results were available from the years prior to initiation of mechanical ventilation. In accordance with national guidelines, mechanical ventilation was electively initiated in case of symptoms of nocturnal hypoventilation and a carbon dioxide (pCO_2_) level   45 mmHg during sleep, or when pCO_2_ reached ≥ 52.5 mmHg during sleep without symptoms [12]. Measurements of capillary pCO_2_ during routine follow-up visits were used to screen for hypoventilation. In general, patients were invited for follow up visits once a year. In case of symptoms of nocturnal hypoventilation (such as morning headache, daytime sleepiness, disturbed sleep, fatigue and impaired concentration) or increased daytime pCO_2_, overnight arterial or transcutaneous measurements were obtained to confirm or exclude nocturnal hypoventilation. In case of transcutaneous pCO_2_ measurement 1 or 2 capillary bloodgasses were obtained to compare to transcutaneous pCO_2_. After diagnosis of nocturnal hypoventilation, admission was planned to initiate non-invasive nocturnal ventilation. In some cases mechanical ventilation was continued after an episode of acute respiratory failure because of surgery or infection.

We determined the presence of homozygous loss of *Survival Motor Neuron 1* (*SMN1)* function and *SMN2* copy number with multiplex ligation-dependent probe amplification (MLPA; SALSA kit P021-B1-01, MRC-Holland) [13]. We used the SMA classification system based on clinical features as described previously with some additions, i.e. SMA type 1c for patients who had learned to roll or lift their head in prone position, SMA type 2a for patients who had learned to sit independently and SMA type 2b for patients who had reached the motor milestones of standing with support [2, 14–16]. We captured patient characteristics as described previously [7, 13, 15]. We used patient data obtained prior to participation in a clinical trial or treatment with SMN protein augmenting drugs (i.e., ‘treatment-naïve’). Parameters of lung function and expiratory muscle strength were measured longitudinally at the department of pulmonology and Center of Home Mechanical Ventilation. Forced Vital Capacity (FVC), Forced Expiratory Volume in 1 s ( FEV_1_), Peak Expiratory Flow (PEF) and Maximum Expiratory Pressure (PE_max_) were measured using Geratherm Spirostik® [6, 7].

We included tests of expiratory muscle strength (PE_max_) because respiratory muscle weakness in SMA is characterized by a rather unique pattern with predominant weakness of (mainly expiratory) intercostal muscles and relative sparing of (inspiratory) diaphragm function [6, 16–18]. Also, based on our previous study, we believe PE_max_ is a sensitive screening parameter to detect respiratory muscle weakness in SMA patients [6].

Lung function tests were performed by a small team of professionals experienced in conducting these tests in children and adults with neuromuscular diseases. All lung function tests were measured and reported according to the European Respiratory Society guidelines [19, 20], in sitting position, without corsets or braces. All studied outcomes were reported as standardized values, according to the Global Lung Function Initiative, with the exception of PE_max_ [21]. Tape-measured arm span was used preferably as a surrogate measure for height in patients unable to stand or with a severe scoliosis [7, 22].

The local Medical Ethical Committee approved this study (09-307/NL29692.041.09) and informed consent was obtained from all participants and/or their parents in case of minors. The reporting.

of this study conforms to the STROBE (Strengthening the Reporting of Observational Studies in Epidemiology) statement [23].

### Statistical analyses

We used all available patient data for analysis. For baseline characteristics, we used descriptive statistics. We used linear mixed-effects models (LMMs), hypothesizing a progressive decline of lung function over time, depending on SMA type. The fixed part of our models contained time (in years prior to the initiation of mechanical ventilation), SMA type, and an interaction term of these two predictors as fixed factors. The random part contained an intercept and slope for time per patient. Using a likelihood ratio test, we evaluated whether the rate of decline was significantly different between SMA types. We used estimated baseline values (i.e., projected y-axis intercepts at t = 0) as surrogates for lung function and expiratory muscle strength at initiation of mechanical ventilation. Additionally, to assess whether there was a non-linear change in lung function and expiratory muscle strength over time, we also fitted non-linear models using cubic splines. We used penalized-likelihood criteria (AIC and BIC) to select the optimal model fits. As we found no significant improvements of the models fits, i.e. no evidence for a non-linear change over time for our outcomes, we here report the findings of our LMMs. We assessed differences in lung function results between patients who electively started mechanical ventilation with patients who could not be weaned off the ventilator after surgery or a respiratory tract infection, using LMMs. We used *R* (v4.0.3 for Windows with RStudio v1.4.1103) for all statistical analyses [24]. The statistical models were fitted using the lmer function of *lme4* (v1.1–27.1) [25]. *Ggplot2* (v3.3.5) was used for all data visualization [26].

## Results

We included 38 patients, most of whom had SMA type 2 (71%). Baseline characteristics are shown in Table 1. Median follow up (prior to initiation of mechanical ventilation) was 7.1 years (IQR: 4.8; 12.7). Median age at initiation of mechanical ventilation was 18.8 years (IQR: 13.2; 30.1). Median capillary pCO_2_ during routine follow-up visit prior to initiation of mechanical ventilation was 46 mmHg (IQR 44–48). Majority of patients reported no symptoms suggestive of nocturnal hypoventilation. Nearly one quarter of patients failed to wean of the ventilator and continued nocturnal ventilatory support after scoliosis surgery (n = 1) or after been admitted because of acute respiratory failure due to a respiratory tract infection (n = 8). All patients who started mechanical ventilation electively, were diagnosed with nocturnal hypoventilation by overnight arterial or transcutaneous pCO_2_ measurements. Patients who started ventilatory support electively had a significant (*p* = 0.019) shorter time interval between last lung function test and initiation of nocturnal ventilatory support (median 0.2 years (IQR 0.1; 0.7) compared to patients who failed to wean after acute respiratory failure (median 0.7 years (IQR 0.5; 5.2).

**Table 1:** Baseline characteristics

SMA type	1c	2a	2b	3a
Number of patients	8	20	7	3
Age (years) at start ventilation	20.45	15.98	24.80	47.75
Median (IQR)	(14.25; 23.60)	(12.86; 27.61)	(16.16; 28.08)	(41.80; 55.20)
Ventilatory support started electively, n (%)	6 (75)	16 (80)	4 (57)	3 (100)
Symptoms nocturnal hypoventilation, n (%)	2 (25)	5 (25)	1 (14)	0 (0)
Years between test and start ventilation	0.4	0.2	4.5	0.6
median (IQR)	(0.1; 1.0)	(0.1; 0.7)	(0.1; 5.8)	(0.2; 0.7)

IQR = Interquartile range; n = number

### Forced vital capacity (FVC)

**Fig. 1:**
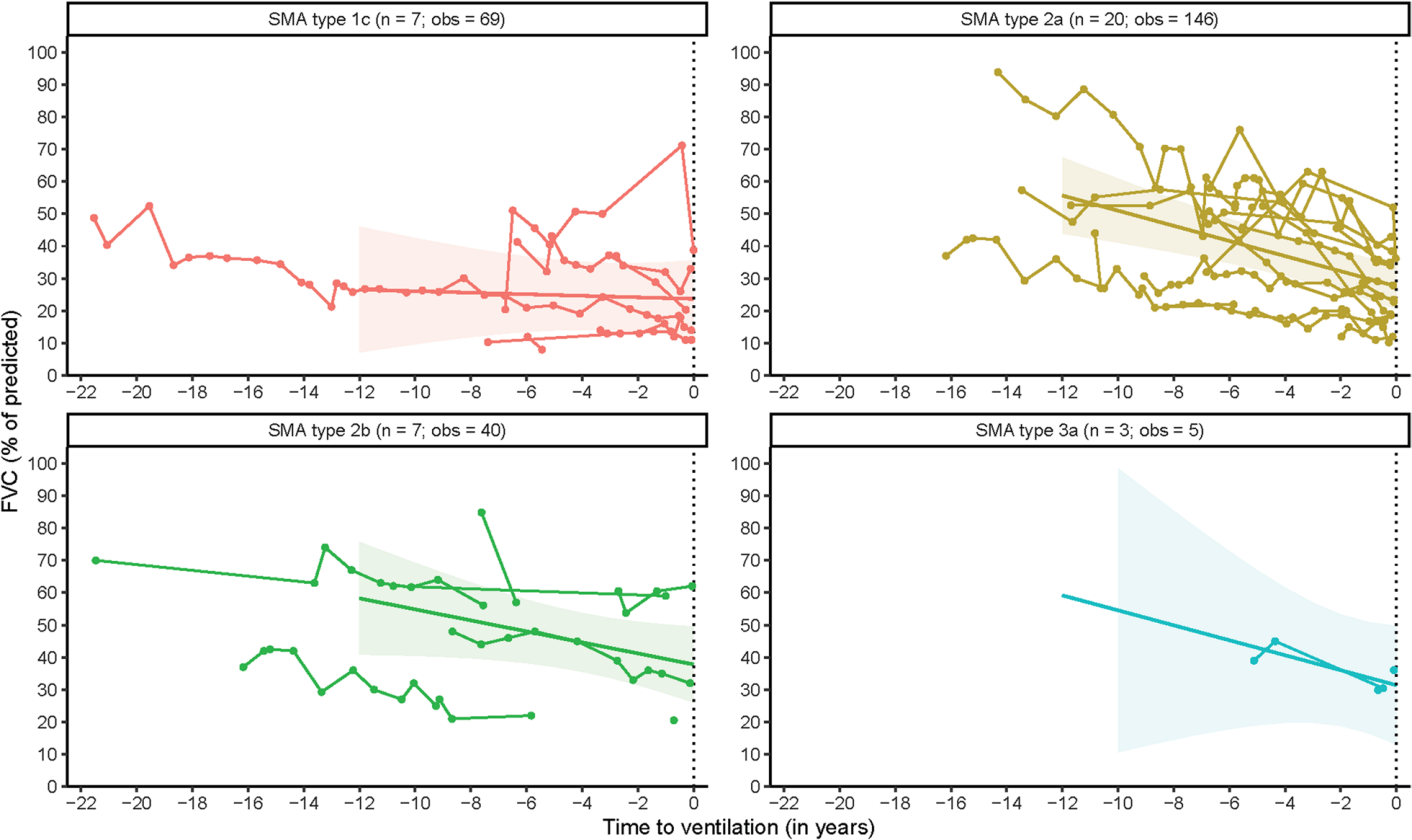
Longitudinal course of Forced vital Capacity (FVC) in the years preceding mechanical ventilation. Legend: time = 0: initiation of mechanical ventilation; n = number of patients; obs = number of observations

In total, 260 FVC measurements of 37 patients were available for longitudinal analyses. Median FVC at initiation of ventilation was 28.84% (95% CI: 23.48; 34.17). The rate of %FVC change over time prior to the initiation of mechanical ventilation averaged − 1.75%/year (95% CI: − 2.66; − 0.86). Our data suggested a stable rate of decline over time, but due to limited data we were unable to reliably assess whether there was an accelerated decline during the 1-year period preceding initiation of mechanical ventilation. FVC was lowest at initiation of mechanical ventilation in the individual patient who electively started mechanical ventilation. Both FVC at initiation of mechanical ventilation and rate of FVC decline preceding ventilation did not differ significantly between SMA types (Table 2, Fig. 1). Estimated median FVC at initiation of mechanical ventilation was significantly higher (*p* < 0.001) in patients who could not be weaned off the ventilator after surgery or a respiratory tract infection (median FVC 38.55%, 95% CI 29.02; 48.08) compared to patients who electively started mechanical ventilation (median FVC 25.28%, 95% CI 19.75; 30.81).

**Table 2:** Lung function test results at start of mechanical ventilation (= intercept) and average annual change in the years prior to start mechanical ventilation (= slope)

Lung function test (LFT)	SMA type	n (obs)	Annual change LFT (SE)	95% CI	LFT at start MV (SE)	95% CI
Standardized FVC (%)	Total group	37 (260)	− 1.75 (0.45)	− 2.66; − 0.86	28.84 (2.69)	23.48; 34.17
	1c	7 (69)	− 0.24 (0.94)	− 1.99; 1.53	23.69 (6.03)	12.19; 34.99
	2a	20 (146)	− 2.34 (0.58)	− 3.42; − 1.26	27.57 (3.54)	20.89; 34.27
	2b	7 (40)	− 1.70 (0.94)	− 3.49; 0.06	37.82 (5.93)	26.80; 49.27
	3a	3 (5)	− 2.31 (2.40)	− 6.81; 2.19	31.40 (9.29)	13.78; 49.03
Standardized FEV_1_ (%)	Total group	38 (385)	− 1.72 (0.34)	− 2.40; − 1.04	28.82 (2.44)	23.99; 33.69
	1c	8 (74)	− 0.27 (0.72)	− 1.62; 1.09	26.10 (5.31)	15.96; 36.15
	2a	20 (243)	− 1.95 (0.43)	− 2.76; − 1.15	27.79 (3.24)	21.67; 33.99
	2b	7 (47)	− 2.15 (0.78)	− 3.62; − 0.67	36.40 (5.79)	25.65; 47.73
	3a	3 (21)	− 2.63 (1.26)	− 5.01; − 0.25	30.72 (8.56)	14.41; 47.01
Standardized PEF (%)	Total group	20 (247)	− 1.65 (0.46)	− 2.59; − 0.71	29.96 (3.31)	23.35; 36.67
	1c	5 (60)	− 0.64 (0.89)	− 2.40; 1.11	33.88 (6.75)	20.78; 47.16
	2a	15 (187)	− 1.99 (0.51)	− 3.00; − 0.97	28.71 (3.91)	21.12; 36.38
PE_max_ (cmH_2_O)	Total group	32 (279)	− 0.03 (0.34)	− 0.78; 0.66	35.30 (2.97)	29.41; 41.36
	1c	6 (55)	0.02 (0.86)	− 1.58; 1.62	25.21 (6.44)	13.22; 37.36
	2a	17 (175)	0.17 (0.44)	− 0.74; 0.99	36.40 (3.89)	29.08; 43.72
	2b	6 (37)	− 0.35 (0.83)	− 1.93; 1.19	41.63 (7.61)	27.43; 55.99
	3a	3 (12)	− 0.13 (1.95)	− 3.85; 3.52	40.03 (11.68)	17.92; 61.99

CI = Confidence interval; FEV_1_ = Forced Expiratory Volume after 1 s; FVC = Forced Vital Capacity; MV = mechanical ventilation; n = number of patients; obs = number of observations; PEF = Peak Expiratory Flow; PE_max_ = Maximum Peak Expiratory Pressure; SE = standard error

### Forced expiratory volume in 1 s (FEV_1_)

**Fig. 2:**
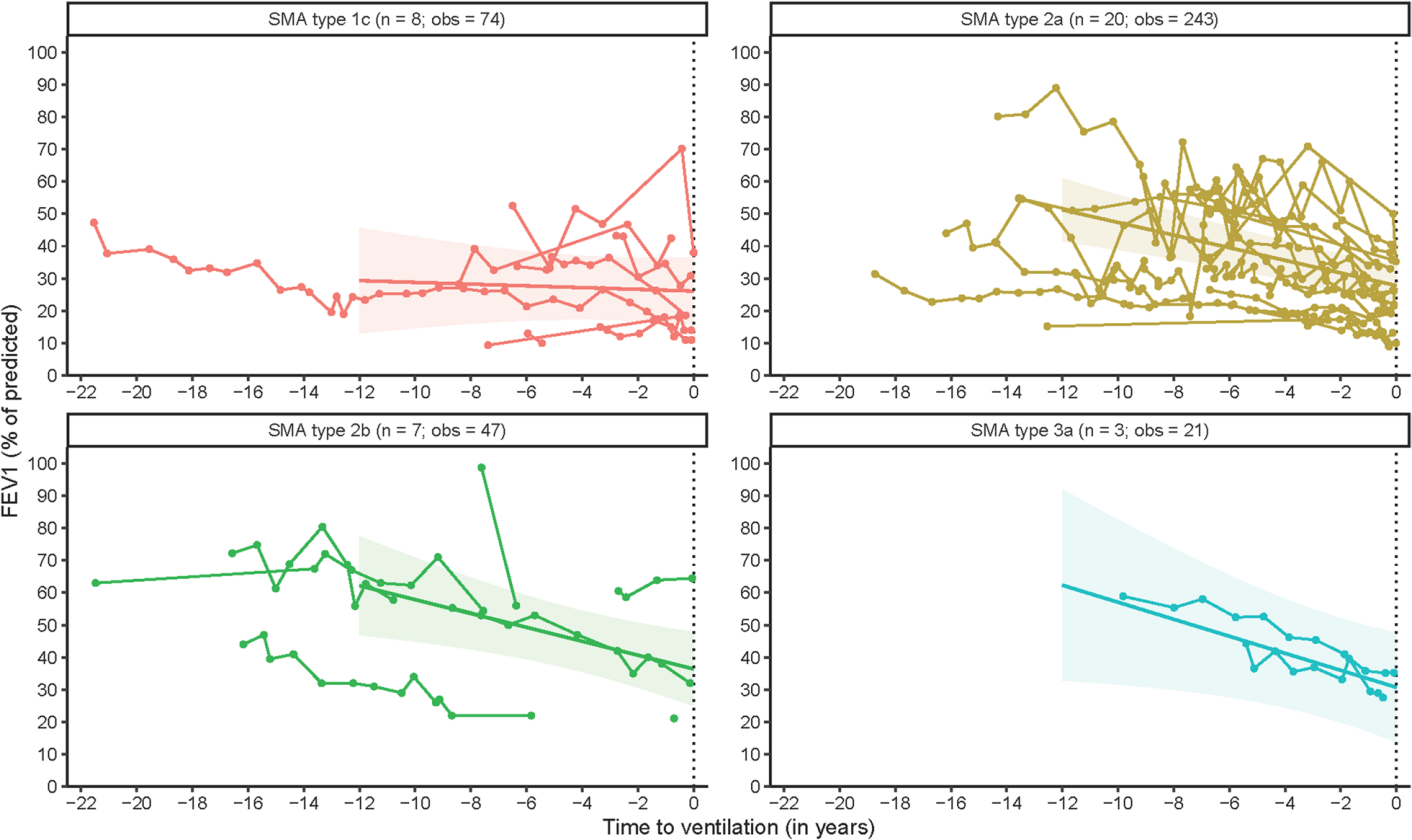
Longitudinal course of Forced Expiratory Volume after 1 s (FEV_1_) in the years preceding mechanical ventilation. Legend: time = 0: initiation of mechanical ventilation; n = number of patients; obs = number of observations

We obtained a total of 385 FEV_1_ measurements from 38 patients for longitudinal analyses. The estimated FEV_1_ at initiation of mechanical ventilation for all included patients was 28.82% (95% CI: 23.99; 33.69). The rate of %FEV_1_ change prior to mechanical ventilation averaged − 1.72%/year (95% CI: − 2.40; − 1.04). As with FVC, we did not observe differences in annual rates of FEV_1_ decline preceding mechanical ventilation between SMA types (Table 2, Fig. 2). Estimated median FEV_1_ at initiation of mechanical ventilation was significantly higher (*p* < 0.001) in patients who could not be weaned off the ventilator after surgery or a respiratory tract infection (median FEV_1_ 39.14%, 95% CI 30.64; 47.63) compared to patients who electively started mechanical ventilation (median FEV_1_ 25.55%, 95% CI 20.37; 30.72).

### Peak expiratory flow (PEF)

**Fig. 3:**
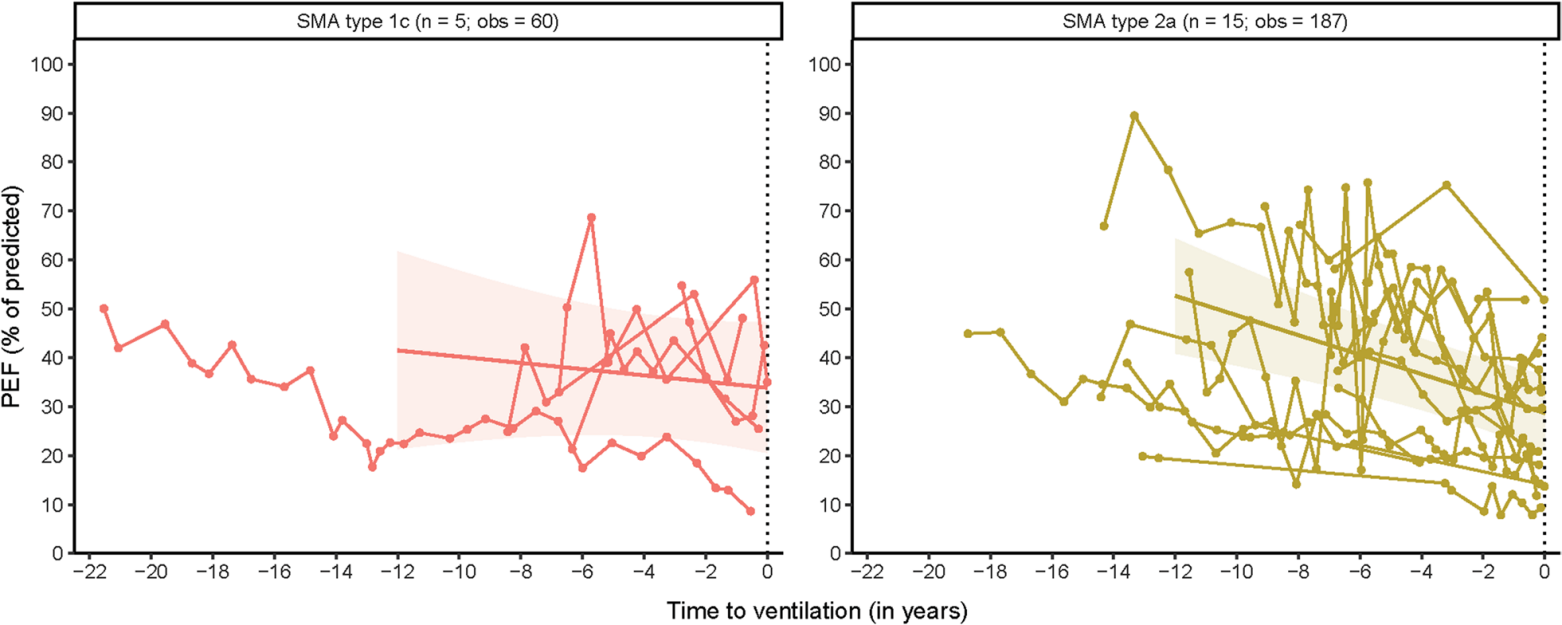
Longitudinal course of Peak Expiratory Flow (PEF) in the years preceding mechanical ventilation. Legend: time = 0: initiation of mechanical ventilation; n = number of patients; obs = number of observations

A total of 247 assessments of PEF from 20 patients with SMA types 1c and 2a, obtained prior to the start of mechanical ventilation, were available for analyses. At initiation of mechanical ventilation median PEF was 29.96% (95% CI: 23.35; 36.67). We found an average annual decline of 1.65%/year (95% CI: 0.71; 2.59). The average change in SMA type 1c (− 0.64%/year) was smaller than in type 2a (− 1.99%/year), but this difference was not significant (Table 2, Fig. 3). Estimated median PEF at initiation of mechanical ventilation was significantly higher (*p* < 0.001) in patients who could not be weaned off the ventilator after surgery or a respiratory tract infection (median PEF 45.33%, 95% CI 33.95; 56.70) compared to patients who electively started mechanical ventilation (median PEF 25.11%, 95% CI 18.85; 31.38).

### Maximum expiratory pressure (PE_max_)

**Fig. 4:**
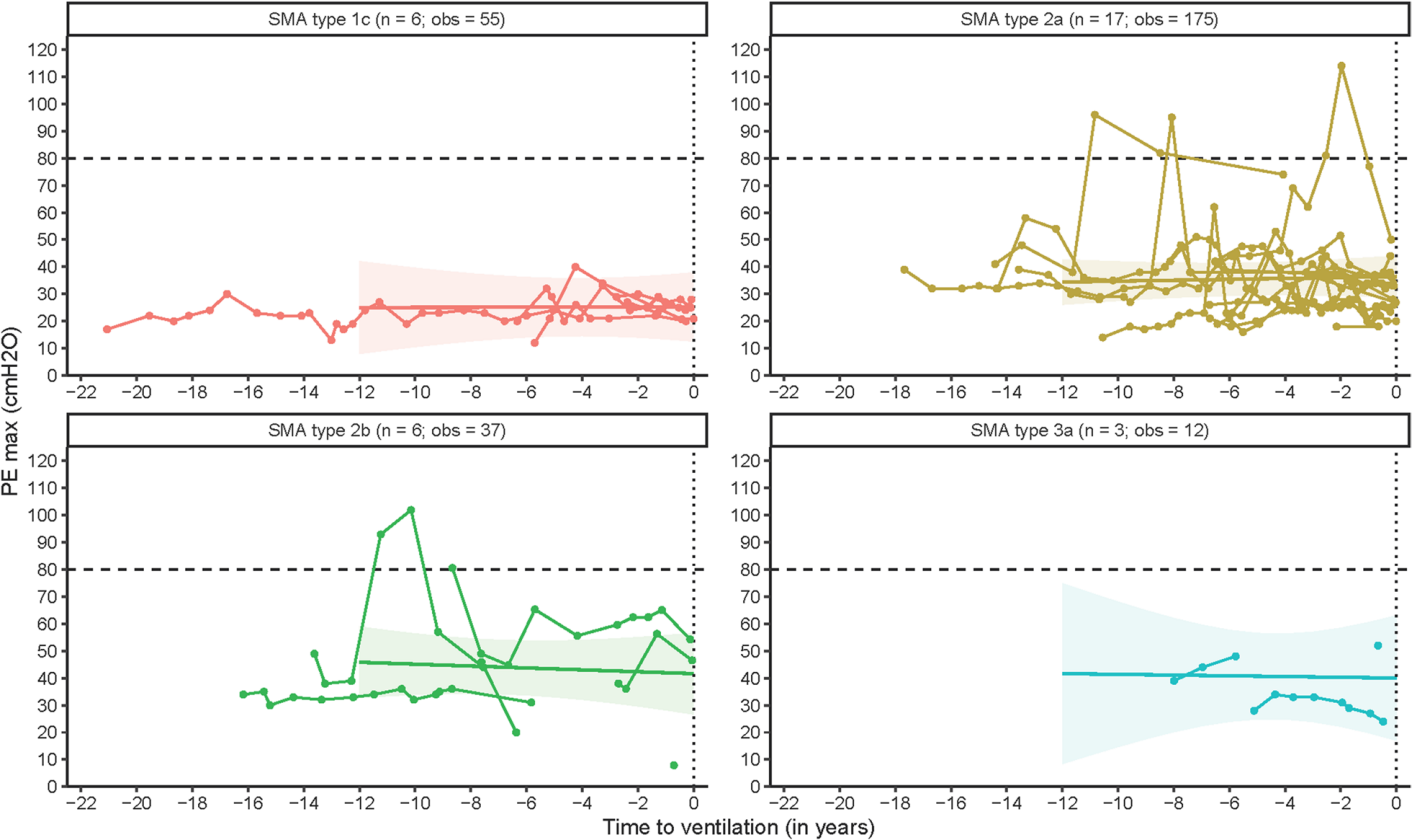
Longitudinal course of maximum peak expiratory pressure (PE_max_) in the years preceding mechanical ventilation. Legend: time = 0: initiation of mechanical ventilation; n = number of patients; obs = number of observations. Dotted horizontal line represents the lower limit of normal

We obtained a total of 279 observations of PE_max_ from 32 patients for longitudinal analyses. Estimated PE_max_ at initiation of mechanical ventilation was 35.30 cmH_2_O (95% CI: 29.41; 41.36), which is far below the lower limit of normal (80 cmH_2_O). In contrast to the other studied outcomes, PE_max_ remained stable in the years preceding mechanical ventilation. There was an average change of − 0.03 cmH_2_O/year (95% CI: − 0.78; 0.66). We did not find significant differences between SMA types (Table 2, Fig. 4). Estimated median PE_max_ at initiation of mechanical ventilation was significantly higher (*p* < 0.001) in patients who could not be weaned off the ventilator after surgery or a respiratory tract infection (median PE_max_ 38.22 cmH_2_O, 95% CI 31.66; 44.79) compared to patients who electively started mechanical ventilation (median PE_max_ 26.04 cmH_2_O, 95% CI 14.94; 37.14).

## Discussion

The natural history of lung function in SMA types 1c-3a is characterized by a decline from an early age. In this study we analyzed the natural course of lung function in the years preceding chronic respiratory failure requiring mechanical ventilation in treatment-naïve SMA patients, in order to identify lung function patterns that predict impending respiratory failure. However, lung function decline rates remained stable prior to respiratory failure. This is in contrast to the previously described accelerated increase in carbon dioxide levels [11].

Both inspiratory and expiratory functions become severely compromised in patients with SMA. At initiation of mechanical ventilation, median FVC, FEV_1_ and PEF scores were around 30% of predicted, while median PE_max_ was 35 cmH_2_O. The average annual decline of FVC, FEV_1_ and PEF in the years preceding initiation of mechanical ventilation was 1.7%. Previous studies also included ventilated patients, which probably at least partly explains the difference to the reported rate of decline of this study [5, 7]. In our previously published national cohort study we found an annual FVC decline of 1.32% in patients with SMA type 2a, 1.4% in SMA type 2b and 0.67% in SMA type 3a [7]. Trucco et al. described an annual decline of 4.2% in patients with SMA type 2 aged 5–13 years, followed by a slower decline of 1% per year and an annual FVC decline of 6.3% in patients with SMA type 3 between 8 and 13 years, followed by a slower decline of 0.9% per year [5]. Although baseline lung function values differed between SMA types, the annual decline rates were not significantly different between different SMA types. This is probably due to the relatively small patient sample, since rates of decline observed in natural history studies over longer periods of time differed between SMA types [6, 7]. In contrast to the other parameters, PE_max_ remained relatively stable but very low in the years prior to mechanical ventilation, indicating that the main loss of expiratory muscle strength occurs earlier in the disease course. We observed lower lung function test results at initiation of mechanical ventilation in patients who electively started mechanical ventilation, compared to patients who could not be weaned off the ventilator after surgery or a respiratory tract infection. The standards of care recommendations on respiratory management of SMA states that there is strong consensus to start mechanical ventilation after documented respiratory failure or hypoventilation during sleep. They also suggest, with less consensus, to initiate mechanical ventilation before documented respiratory failure, to palliate dyspnea in non-sitters or when there are recurrent respiratory tract infections in both non-sitters and sitters [1]. This study does not gather new evidence to determine if early initiation of mechanical ventilation, i.e. prior to occurrence of hypoventilation during sleep, is better.

There are only a few studies that have analyzed lung function in patients with SMA and, to the best of our knowledge, none focused specifically on the period preceding chronic respiratory failure, which necessitates initiation of mechanical ventilation [3–5, 7, 27–30]. Bach et al. described a correlation between vital capacity and definitive dependence on continuous mechanical ventilation in patients with SMA type 1 and 2. However, the relation between lung function decline and initiation of mechanical ventilation was not studied as non-invasive ventilation during sleep was already prescribed from the time of diagnosis in all patients SMA type 1 and 2a with paradoxal breathing [27]. Most previous studies have used FVC to study lung function [3, 5, 31, 32]. Median FVC before the start of mechanical ventilation in our study was in line with some previous findings, e.g. of a mean FVC of 30% at initiation of nocturnal non-invasive mechanical ventilation in 11 patients with SMA type 1c and 34 with type 2 [3]. However, other studies have reported slightly different results. A recent study reported a higher median FVC (44%, IQR 28.5–57) in 55 treatment-naïve patients with SMA type 2 at the start of mechanical ventilation. This difference is probably explained by the younger median age (5 years, range: 1.8–16.6) in comparison to our study population [5]. Another retrospective study reported a much lower FVC of < 20% at initiation of mechanical ventilation in 4 patients with SMA type 2 [31]. The small sample size or possible selection bias may have caused the differences in comparison to our findings. This may be further illustrated by a study that reported comparable median FVC outcomes to our findings (i.e. 31.5%, range 11.3–82.8) in 11 patients with SMA type 2 with a median age of 25.8 years who did not use mechanical ventilation. Three patients even had an FVC < 30% [32]. We cannot exclude the possibility that these patients eventually started mechanical ventilation after publication of these studies. Despite the scarcity of data and the obvious differences between studies, recent standards of care indicate that FVC values < 40% are associated with an increased risk of (N)REM-related sleep disordered breathing, requiring mechanical ventilation [1, 9]. Our data suggest that the predictive value of lung function tests may also have its limitations.

Data on other lung function parameters than FVC are even more scarce. Lyager et al. reported a median FEV_1_ of 31.6% (range 22.4–87) and a median PEF of 41.6% (range 23.7–96.6) in respectively 11 and 10 non-ventilated patients with SMA type 2, but did not report if these patients eventually started mechanical ventilation [32]. More recently, a few studies documented on the longitudinal course of lung function in treatment-naïve SMA patients, but they did not address the relationship between lung function decline and the initiation of mechanical ventilation [4, 5, 7, 27–30]. Gilgoff et al. were, to the best of our knowledge, the only to report longitudinal data from the years preceding initiation of mechanical ventilation, but with only 8 observations obtained from 4 patients [31].

Our and other data showed a decline of lung function in treatment-naïve patients with SMA type 1c–3a, which is most pronounced in childhood. Lung function did reach a plateau in early adulthood, while patients with late onset SMA (types 3b and 4) are likely to have a stable lung function throughout life [7]. Of note is that the varying inclusion of both ventilated and non-ventilated patients in previous studies may partially explain the differences in observed rates of decline. The observed lung function values described here, in treatment naïve patients during the years preceding initiation of mechanical ventilation, may therefore reflect the range in which respiratory reserve capacity is likely to be exhausted. Our longitudinal analysis suggests that the previously described natural course of decline does not accelerate prior to the start of mechanical ventilation. This is in contrast to our previously reported accelerated increase in pCO_2_ values in the years prior to start of mechanical ventilation [11].

The ability to predict the need for mechanical ventilation is important to ensure that patients will not be confronted with emergency decision regarding the start of ventilation. The current data will therefore not help to improve counseling of patients about the best timing for interventions that could prevent or treat respiratory failure, in contrast to previously described pCO_2_ levels [11].

Our work has important strengths. First, we provided longitudinal data in non-ventilated SMA patients not only for FVC, but also for FEV_1_, PEF and PE_max_. This is the first study describing the course of lung function with a full range of lung function tests prior to start of mechanical ventilation in a relatively large cohort. Secondly, the cohort of genetically and clinically well-defined patients with SMA with a relatively large number of repeated measurements and long follow-up allowed for more detailed longitudinal analyses.

We also acknowledge limitations of our work. The sample size limits the power of this study. Limited data were available in the year preceding mechanical ventilation. For this reason we were unable to reliably compare the rate of lung function decline the year preceding mechanical ventilation to the years before. Nevertheless, our cohort was sufficiently large to model the natural history in the years preceding respiratory failure in different types of SMA.

The age at which mechanical ventilation was initiated was relatively old, especially in patients with SMA type 1c. This is explained by the study design, since we only included patients who performed lung function testing prior to initiation of mechanical ventilation [8]. Also, the number of patients with SMA type 1 was limited, as most patients with SMA type 1 require nocturnal ventilation at a young age, i.e. prior to their ability to perform reliable lung function testing. We only included a limited number of patients with SMA type 3, as respiratory failure is rare in this group [8]. However, these data are important, as very limited data are available on lung function and respiratory failure in the milder SMA phenotypes. Our study lacks an assessment of possible confounders, such as severity of (corrected) scoliosis, use of airway clearance techniques and nutritional status. However, we consider this less important as our study focuses on the natural history of SMA with treatment according to the standards of care [1, 16]. In this study we did not compare these lung function data to SMA patients with similar lung function who did not yet require mechanical ventilation. Finally, some of the patients had important test to test variation in lung function, at least partly explained by respiratory muscle fatigue which is common in patients with SMA [33], and by the surrogate measures of height used to determine the standardized lung function values.

## Conclusions

This study is the first study to describe the longitudinal course of lung function in the years preceding chronic respiratory failure necessitating mechanical ventilation in a cohort of treatment-naïve SMA patients. The deterioration of lung function does not accelerate prior to the start of mechanical ventilation and therefore patterns of lung function decline do not help to improve counseling of patients about the best timing for interventions that could prevent or treat respiratory failure.

## Data Availability

The datasets used and/or analyzed during the current study are available from the corresponding author on reasonable request.

## Abbreviations

CI: Confidence interval
FEV_1_: Forced expiratory volume in 1 s
FVC: Forced vital capacity
IQR: Interquartile range
LMM: Linear mixed-effects model
MV: Mechanical ventilation
MLPA: Multiplex ligation-dependent probe amplification
N: Number
Obs: Observations
PE_max_: Maximum expiratory pressure
PEF: Peak expiratory flow
pCO_2_: Carbon dioxide
SE: Standard error
SMA: Spinal muscular atrophy
SMN: Survival motor neuron
STROBE: Strengthening the reporting of observational studies in epidemiology
